# Taxonomic and functional diversity of land snails reflects habitat complexity in riparian forests

**DOI:** 10.1038/s41598-023-36896-6

**Published:** 2023-06-17

**Authors:** Voichița Gheoca, Ana Maria Benedek, Erika Schneider

**Affiliations:** 1 Faculty of Sciences, Applied Ecology Research Center, Lucian Blaga University of Sibiu, 5-7 Raţiu Street, 550012 Sibiu, Romania; 2 Department Aueninstitut/Institute for Wetlands Ecology, Institute for Geography and Geoecology, KIT ‒ Karlsruhe Institute for Technology – University of Land Baden-Württemberg and Research Center of the Helmholtz Society, Josefstrasse 1, 76437 Rastatt, Germany

**Keywords:** Biodiversity, Community ecology, Riparian ecology

## Abstract

Habitat complexity affects the structure and dynamics of ecological communities, more often with increased complexity leading to greater species diversity and abundance. Among the terrestrial invertebrate groups, the low vagility of land snails makes them susceptible to react to small-scale habitat alteration. In the current paper we aimed to assess the relationship between taxonomic and functional composition and diversity of land snail communities and habitat structure in the riparian forest habitat. We found that both snail abundance and species richness responded positively to the increase in habitat complexity. The complexity of the riparian forest affected also the snail trait composition. Forest species, species living in woody debris, leaf litter, and root zone and those feeding on detritus were more abundant in complex habitats, while large snails with more offspring, snails having the ability to survive longer periods of dryness, as well as species that prefer arid habitats, were more abundant in less complex habitats. We concluded that habitat complexity promoted functional diversity, with the amount of woody debris as main positive driver, and the adjacent agricultural fields as negative driver of functional diversity.

## Introduction

Habitat structure corresponds to the presence of physical elements in an ecosystem and is one of the most important ecological features that influences patterns and processes of biological communities. It generally refers to the geometry of the physical habitat, including the bare substrate itself (e.g., rock, soil, sediments) and the structure provided by the species that characterize that habitat (e.g., trees, macrophytes, corals, oysters)^1,2^.

One of the foundational theories in community ecology, the habitat heterogeneity hypothesis proposes that an increase in the number of habitats leads to a more diverse species assemblage^3,4^. The literature abounds in studies conducted in the last sixty years with examples of complex habitats sheltering larger number of species than less complex ones. This pattern was documented in terrestrial habitats for a wide range of taxa, including mammals^5^, birds^6^, reptiles and amphibians^7^, arthropods^8^.

Although several studies reported a negative relationship between environmental heterogeneity and species diversity^9–11^, a recent global meta-analysis found that their overall relationship across taxa and spatial scales is positive, as predicted by the ecological theory^12^.

Despite the large number of studies, there is no universally accepted definition of habitat complexity^2,13–15^. The lack of agreement emerges partly because of the impossibility of reaching a consensus regarding the importance of different dimensions of complexity. A solution seems to be to develop quantitative metrics capturing aspects of complexity that are important to organisms, instead of trying to assess the entire phenomenon^2^. This approach allows the selection of a subset of core features that can be taxa-specific.

Riparian ecosystems, developed along river valleys at the interface between terrestrial and freshwater ecosystems, are crucial for landscape-level biodiversity, especially in highly anthropic and agricultural areas. Their contribution to both landscape and biodiversity is disproportional to the relatively reduced coverage^16^. Riparian forests are one of the most complex ecological systems in the world and play an important role in preserving the river and landscape vitality and serve as corridors for maintaining regional biodiversity^17^. The main structural elements of a riparian forest consist in the shoreline vegetation cover and the morphology of the river channel^17^. The vegetation reduces the insulation, evaporation, and velocity of runoff water, providing time for the water to infiltrate into the soil^18^. The generated leaf litter and the root zone support a higher microbial community diversity, enhancing the decomposition of organic matter^19^. The leaf litter and plant debris also shelter rich invertebrate communities having a significant contribution to the global biodiversity^20^.

The vicinity of the river and the groundwater discharge, common in the riparian zones, makes this area a potential key habitat for forest snails^21^. Also, many land snails are litter-dwellers, depending on moist litter with relatively high calcium content^22,23^. Although snails are not major decomposers in many ecosystems, they can easily be monitored and due to their low vagiliy and microhabitat specificity are suitable as model organisms to better understand the way other terrestrial invertebrates could respond to changes in riparian forest structure^24^.

While the taxonomic diversity (species richness) remains the main measure of biodiversity, functional trait-based approaches are gaining field. Using traits allows the characterization of organisms in terms of the biological attributes responsible of their functional responses to the abiotic and biotic environment^25^. A large number of studies use trait-based approaches to functionally link individual organisms to community structure and dynamics.

There are numerous studies that have examined the effects of habitat complexity on species composition and diversity of terrestrial snail communities [e.g.,^26–29^]. Other studies have used a trait-based approach in order to assess the effect of environmental components on the trait composition in land snail communities, or the snail importance as decomposers^30–32^. To our knowledge, no attempt was made to link land snail traits to habitat complexity at community level. Therefore, we are addressing for the first time the relationship between land snail community trait composition and diversity and the complexity of a particular habitat type. Our study aims to assess the responses of land snails of an increasingly threatened ecosystem, the riparian forests, to changes in habitat structure. We tested the following hypotheses: (1) complex riparian habitats shelter snail communities with higher abundance and species richness, and a different species composition than less complex ones; (2) high habitat complexity sites have different trait composition of snails from low habitat complexity sites; (3) functional diversity of snails is positively associated with habitat complexity.

## Material and methods

### Study area and habitat type

**Figure 1:**
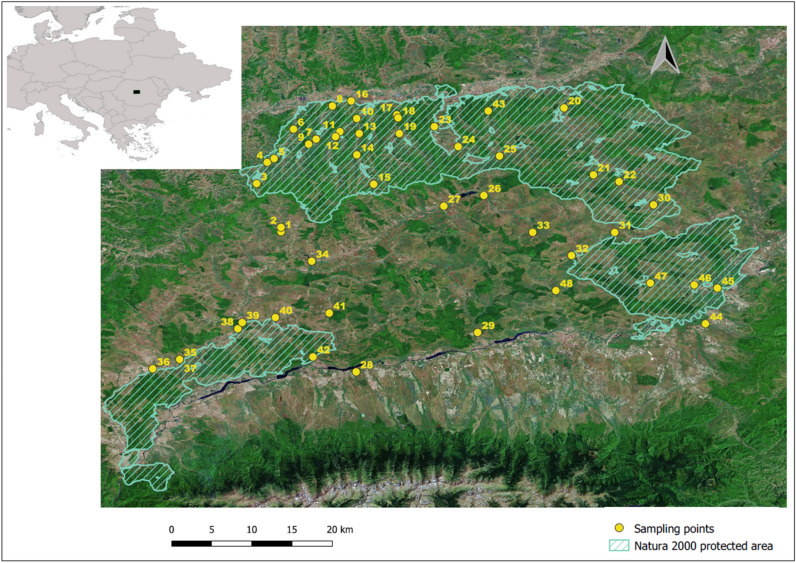
The location of the sampling points in the studied area. The map was made in QGIS version 3.16^34^.

The field survey was conducted in 2017–2018. We studied 48 riparian habitat forests of 91E0 Natura 2000 habitat type, subtype 44.13 Salicion albae, located in the southern area of the Transylvanian Plateau, Central Romania (Fig. 1). Most of the selected forests are part of the Natura 2000 European network of protected areas, being included in three sites: ROSCI0227 Sighișoara Târnava Mare, ROSCI0303 Hârtibaciu Sud-Est, and ROSCI0304 Hârtibaciu Sud-Vest.

The riparian willow forests in the study area are constructing primarily well-structured compact galleries along the river's courses, with three to five-layered forests with a tree canopy dominated by *Salix alba* and *S. fragilis* in various proportions. The canopy layers are connected by lianas that form thick covers on trees and shrubs. In some short river sectors, with deep valleys and steep slopes that maintain a cooler microclimate, the forests belong to subtype 44.2. (Alnion incanae), with *Alnus incana* and other montane species, such as the tall herb *Telekia speciosa*. The area is a rural one with various agricultural and zootechnical practices, as well as clandestine logging by the riverine population, affecting riparian habitats.

### Sampling sites and method

The sampling sites were located on Târnava Mare, Hârtibaciu, and Olt rivers and their affluents (Fig. 1 and Appendix A, Table S1).

At each sampling site, land snails were visually searched for in a 100 m^2^ quadrat by two persons for one hour in all microhabitats suitable for snails^33^. In addition, in each site, a 20 l sample of litter and topsoil was sieved through a 10 mm mesh sieve, the resulting material was bagged and taken to the laboratory. After drying and further sieving, the material was searched for snails directly or using a binocular microscope, depending on their size. Only live specimens and fresh empty shells with intact periostracum were considered in the analyses.

### Habitat complexity

Habitat complexity was assessed based on ten parameters, selected considering the biology and ecology of land snails. They included the topographic complexity (the presence of terraces), vegetation structure (tree, shrub, tall herb, herb, and liana cover), number of tree species, amount of logs and litter, and destination of neighbouring fields (Table 1). The abundance of decaying wood was estimated by counting the number of dead trees in the sampled area. We noted with (0)- site with no decaying wood, (1)—site with some decaying branches or bark, (2)—site with at least one decaying trunk, and (3)—site with more than one decaying trunk. To estimate the abundance of leaf litter, four random quadrats of 0.25 m^2^ were selected within each sampling plot and the litter was collected, and measured using the cylinder of the sieve.

**Table 1:** Parameters used for habitat complexity assessment.

Habitat characteristics	0	1	2	3
Tree cover (percent)	0–10	11–20	21–50	> 50
Shrub cover (percent)	0–10	11–20	21–50	> 50
Tall herbs (percent)	0–10	11–20	21–50	> 50
Herbaceous vegetation (percent, excluding tall herbs)	0–10	11–40	41–70	> 70
Liana cover (percent)	0–10	11–20	21–50	> 50
Logs	No decaying wood	Some decaying branches	One decaying trunk	>One decaying trunk
Litter (percent of the maximum amount)	0–10	11–40	41–70	> 70
Terraces	No terrace	Terrace on one river bank	Terraces on both riverbanks	–
Neighbouring fields destination	Agricultural	Meadow	Ruderal	Forest
Number of tree species	Quantitative assessment			

The vegetation was considered as the most important feature in assessing habitat heterogeneity in most of the studies regarding riparian forests^7,35,36^. The riparian forest in the area of study is a multilayer forest, each layer contributing to the vertical structure of the forest, maintaining the shade and humidity, and supplying the litter layer, crucial elements for the land snail presence and abundance.

The vegetation cover was assessed by phytocoenological relevés on an area of about 200 m^2^ in each sampling site, and the abundance of plant species was estimated using cover percentages. The evaluation was carried out directly on the field, without collecting any plant material. The species were divided into life-form categories—trees, shrubs, tall herbs, herbs, and lianas—and their total cover was calculated by adding up the cover percentages of individual species. Each parameter was quantified by assigning a number from 0 to 3, except the presence of terraces which was classified from 0 to 2, and the number of tree species, quantitatively assessed (Table 1). The habitat complexity in each sampling site was calculated as the sum of these numbers, ranging from 5 to 24 (Table S1 in Appendix A).

### Snail traits

To characterize the functional features of land snails, we compiled a database containing 13 functional traits (with 54 categories, most of them ordinal), belonging to four groups: morphology and size, reproduction features, specialization, and environmental tolerance (Table 2). The categories were established using Falkner et al.^37^, and they are similar to those used in other studies^30,32^. The database was used to calculate a single value for each trait and species by summing up the relative affinities times the category number^32^. For two traits, the reproduction mode and soil preference, each with two non-mutually exclusive categories, we calculated the percentage of self-fertilization and the percentage of affinity for non-calcareous soil respectively. The habitat, microhabitat, and food preferences were used as independent traits in the analyses.

**Table 2:** Selected snail traits and their values in the original database^37^. In brackets are given the abbreviations used in different graphic representations.

Trait group	Trait	Categories in the database
Morphology and size (Morph)	Shell size (Shsize)	1 < 2.5 mm; 2: 2.5–5 mm; 3: 5–15 mm; 4: > 15 mm
	Shell shape (Shshp)	1: depressed; 2: globose/conical; 3: oblong
Reproduction (Repr)	Sexual maturity (Sexmat)	1: < 1 year; 2: 1 year; 3: > 1 year
	Reproduction mode (Rmode)	1: cross-fertilization, 2: self-fertilization
	Main reproduction periods (Rper)	1: January–February; 2: March–April; 3: May–June; 4: July–August; 5: September–October; 6: November–December
	Number of offspring/eggs per clutch (Nroff)	1: 1–10; 2: > 10
Specialization (Spec)	Habitat preference	woods (woods), shrubs (shrubs), wet woods (wetfor), alluvial forests (alluvfor), open/tall herbs (tallherb), grasslands (grass), water edges (watedge)
	Microhabitat preference	trees/shrubs (tr/shr), herbs (herb), leaf litter (leaflitt), woody debris (woodeb), herb litter (herblitt), soil (soil), root zone (root)
	Soil preference	calcareous (calc), non-calcareous (noncalc)
	Food preference	litter (litter), detritus (detrit), fungi (fungi), lichen (lichen), algae (algae), living vascular plants (livepl), dead vascular plants (deadpl), carnivorous/saprophagous (carn/sap)
Tolerance (Tol)	Humidity preference (humpref)	1: dry/xerophilous; 2: moist /mesophilous; 3: wet/hygrophilous
	Inundation tolerance (inundtol)	1: low; 2: moderate; 3: high
	Dry period survival (drysurv)	1: days; 2: weeks; 3: months

### Data analysis

To evaluate the effect of habitat complexity and its components on the land snail community structure and functions we considered several parameters as response variables. Total abundance was the number of individuals of all species counted in a sample. Species richness was the number of species identified in a sample. The functional diversity, calculated for each trait group (morphology, reproduction, tolerance, and specialization), was expressed as the Rao’s quadratic entropy^38^, which is a generalization of the Simpson diversity index. The relationship between diversity (species richness and Rao’s quadratic entropy) and habitat complexity and its components was analyzed using linear regression models (LM). We chose the best model by stepwise forward selection, based on the F test. In the case of abundance, the assumption of homoscedasticity of residuals was not met; therefore, we accounted for overdispersion using the negative binomial generalized linear model (GLM) function (glm.nb) in MASS package^39^ in R software^40^ and used the χ^2^ test in the stepwise selection. In the best GLM models we evaluated the significance of predictors by comparing the models with and without each of them using the likelihood ratio test of nested models by applying the function lrtest in lmtest package^41^, and the t test in LMs.

In the multivariate analyses, the structural response variables were the species abundances within the community, referred to as species composition hereafter. To evaluate the functional responses of snail communities to habitat complexity we adopted the community-based approach, including the community weighted means (CWM) as response variables, predicted by the environment^42^. To evaluate the response of land snail species composition and functional structure and diversity to habitat complexity, we used the multivariate linear redundancy analysis (RDA), performed in Canoco 5.12 software^43^. We used the interactive forward selection to identify the predictors that best explained the variation in snail community structure or diversity. We corrected the type-I error inflation caused by multiple testing, calculating the adjusted probabilities using the false discovery rate values^42^. We tested the significance of ordination axes by the Monte-Carlo permutation test with 999 unrestricted permutations per test. The significance of response (either positive or negative) to individual predictors was evaluated visually, constructing the t-value biplots with van Dobben circles^42^.

## Results

### The relationship between abundance and taxonomic diversity and habitat complexity

**Figure 2:**
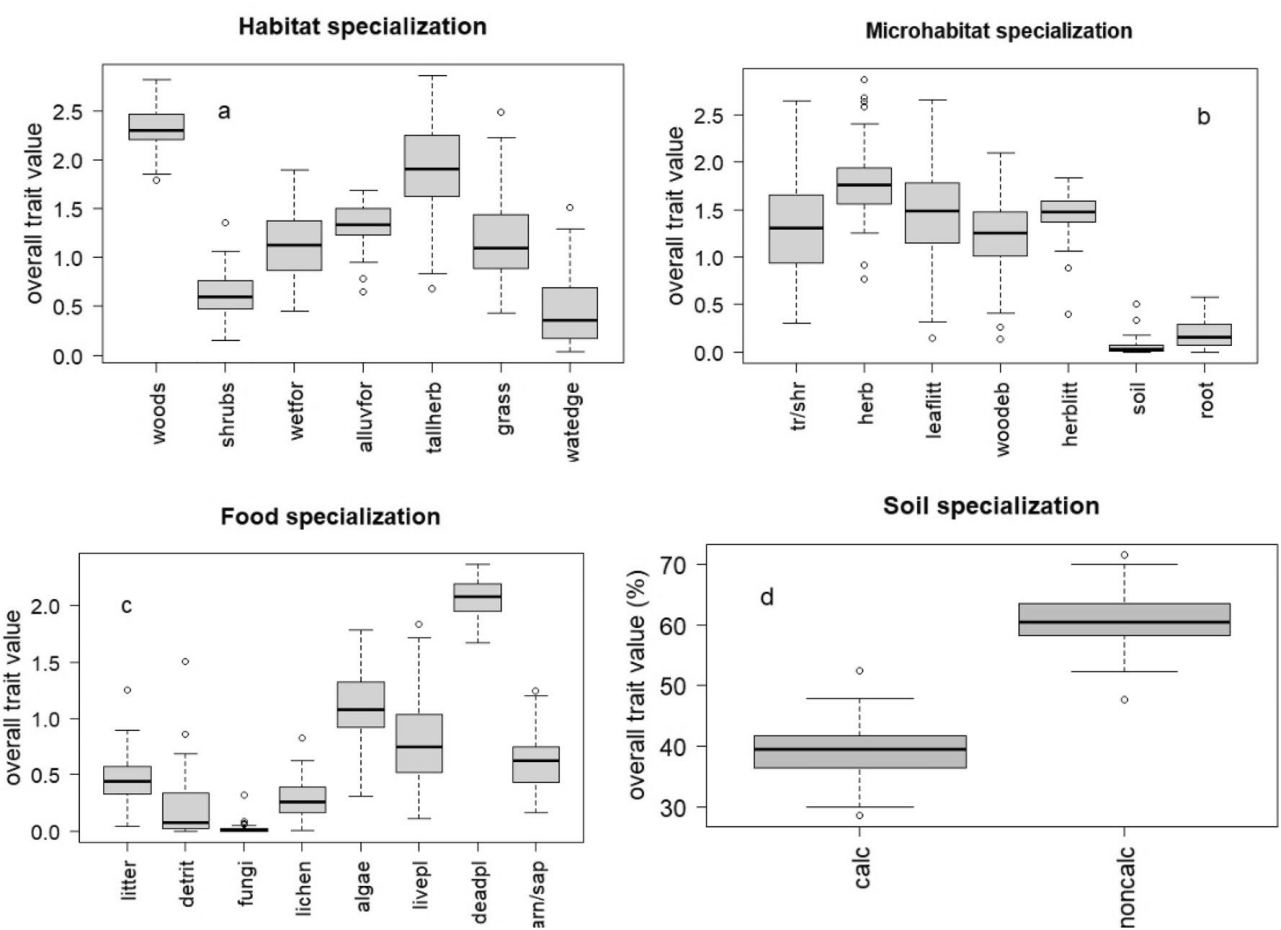
Distribution of snail (**a**) habitat, (**b**) microhabitat, (**c**) food, and (**d**) soil preferences (for categories and abbreviations see Table 2).

We sampled 12,570 land snails of 71 species (Appendix B), including four xeric species (*Cecilioides acicula, Chondrula tridens, Granaria frumentum,* and *Monacha cartusiana*), represented by shells flushed away, therefore they were excluded from the analyses. The abundance of the other species varied between 48 and 947 specimens per sample (mean = 260.5, SE = 31.5). The riparian land snail communities were dominated by forest and tall herb species (Fig. 2a), preferring microhabitats of herbs and litter (Fig. 2b). Most of the snails were consumers of dead and living vascular plants, and algae (Fig. 2c) and had affinity for non-calcareous soils (Fig. 2d).

**Figure 3:**
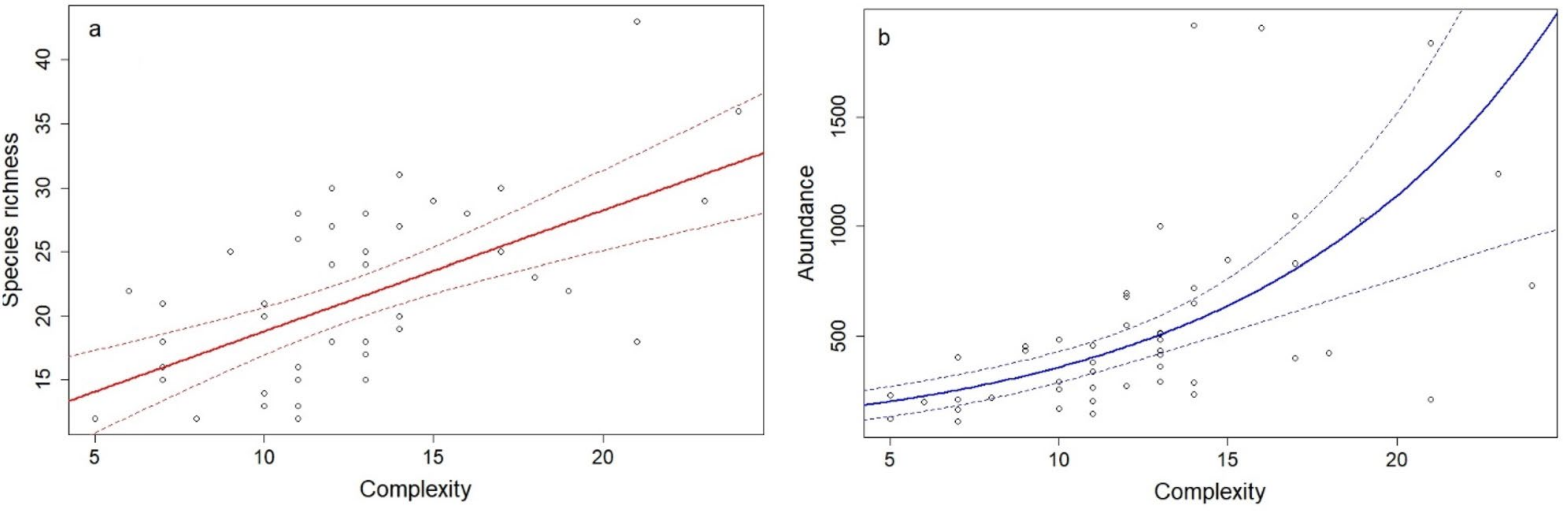
Generalized linear model illustrating habitat complexity as the predictor of (**a**) land snail species richness (gaussian distribution) and (**b**) abundance (negative binomial distribution). The 95% confidence interval for the mean estimated value of species richness and abundance is plotted in dotted lines.

Habitat complexity had a significant positive effect on species richness (t = 5.15, df = 46, p < 0.001, Fig. 3a) and snail abundance (χ^2^ = 29.1, df = 1, p < 0.001, Fig. 3b). Habitat complexity may explain 35.2% of the variation in the snail species richness. Species richness increased by 0.94 species with the increase in complexity with one unit, while total abundance increased 1.12 times. Among the components of habitat complexity, species richness was best predicted by the abundance of logs and tall herb cover (F = 15.3, df = 2 and 44, p < 0.001), which may explain 38.3% of the variation in species richness. Species richness increased by 4.36 species with the increase in logs abundance with one unit (χ^2^ = 18.4, df = 1, p < 0.001, Appendix A, Fig. S1a) and by 2 species with a one unit increase in tall herbs (χ^2^ = 8.5, df = 1, p = 0.003, Appendix A, Fig. S1b). For the total abundance best predictors were litter and terrace (χ^2^ = 34.8, df = 2, p < 0.001), increasing 1.5 times with the increase in litter abundance with one unit (χ^2^ = 21.8, df = 1, p < 0.001, Appendix A, Fig. S1c) and 1.34 times with a one unit increase in terrace (χ^2^ = 7.5, df = 1, p = 0.006, Appendix A, Fig. S1d).

### Species’ responses to complexity

**Figure 4:**
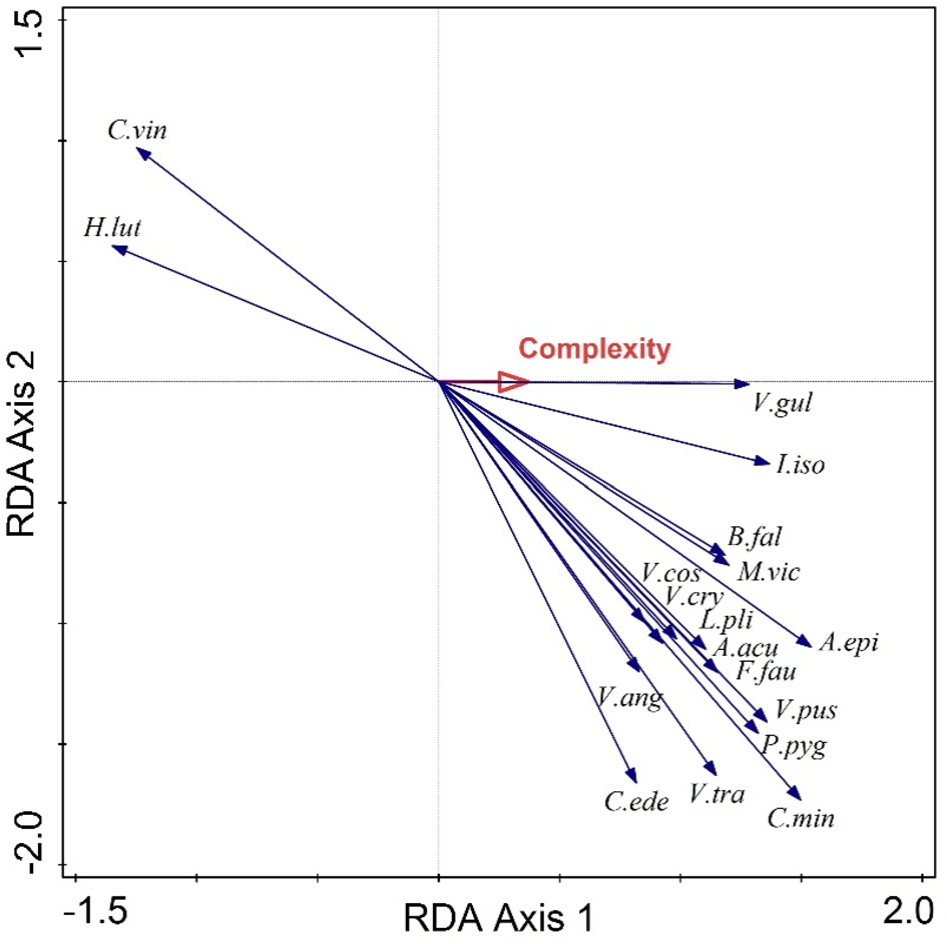
Redundancy analysis (RDA) land snail species-habitat complexity biplot. Only the 18 species with significant response are represented. Species are coded using the initial of the genus name and the first three letters of the species name.

Land snail species composition responded significantly to habitat complexity (pseudo-F = 7.8, p = 0.001), which explained 13.6% (11.7% adjusted) of the variability in species abundance. Most species responded positively to the habitat complexity, being more abundant in complex habitats; for 16 species this response was significant (Fig. 4). Among these species are *Vestia gulo, Isognomostoma isognomostomos, Monachoides vicinus, Balea fallax, Laciniaria plicata, Faustina faustina, Aegopinella epipedostoma, Vallonia costata, Carichium minimum.* In contrast, only two species responded negatively, namely *Helix lutescens* and *Caucasotachea vindobonensis.*

### Snail traits and habitat complexity

**Figure 5:**
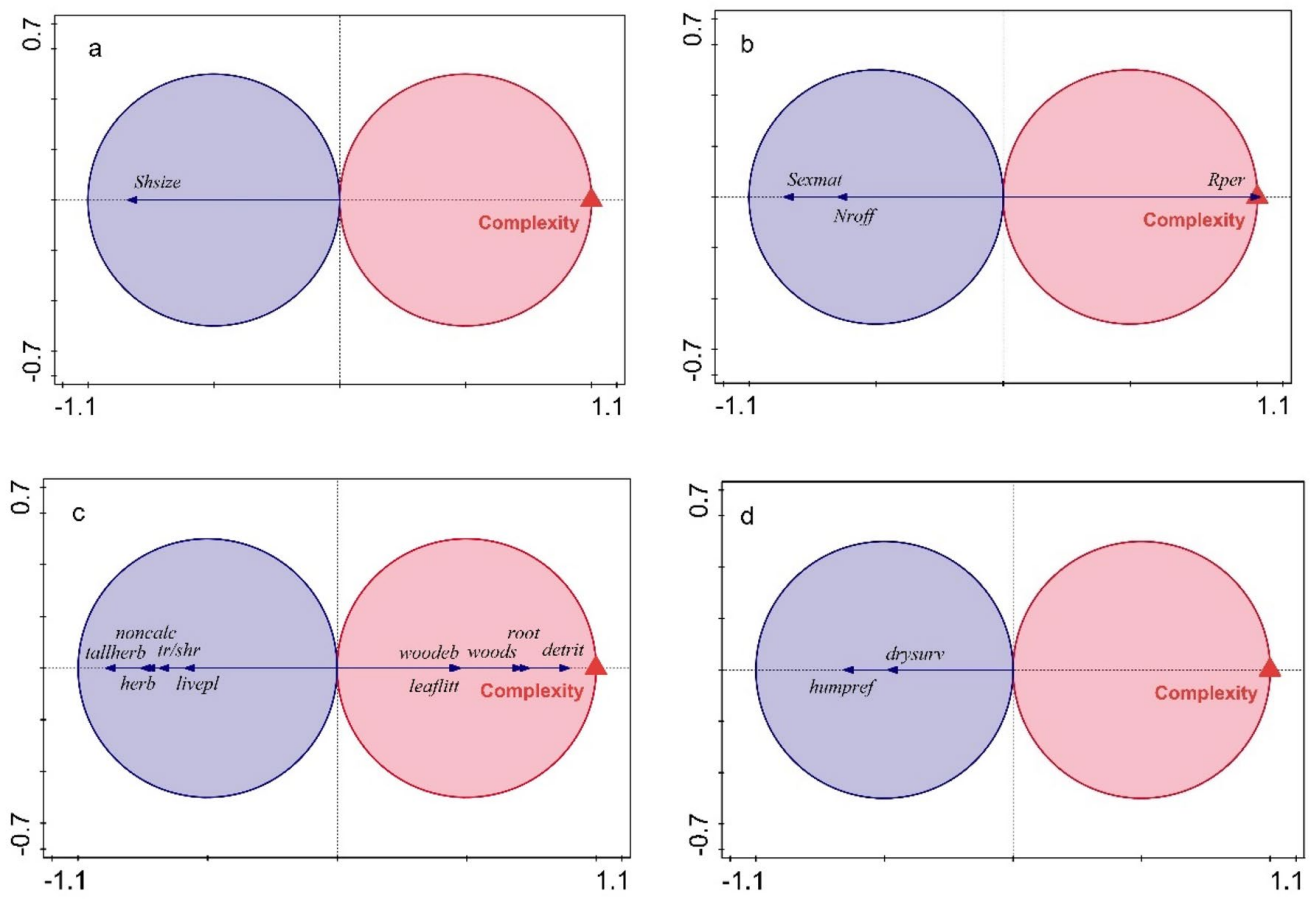
t-value biplots with van Dobben circles in the RDA ordination space illustrating the significance of responses to habitat complexity of the traits concerning (**a**) morphology and size, (**b**) reproduction, (**c**) specialization, (**d**) tolerance for habitat conditions. Traits represented by arrows having their tips in the pink circle show a significant positive response and traits represented by arrows having their tips in the blue circle show a significant negative response. Abbreviations of traits are given in Table 2.

Habitat complexity significantly affected the trait composition of the land snail communities. Snail morphology (shell size and shape) responded significantly to habitat complexity (pseudo-F = 4.2, p = 0.02), which explained 8.4% (6.4% adjusted) of the variation in the community trait composition. In complex habitats best represented were snails with large and oblong shells, but only the response of shell size was significant (Fig. 5a). Most of the reproduction traits responded significantly to habitat complexity (pseudo-F = 5.1, p = 0.012), which explained 10.8% (8.8% adjusted) of the variation in the community trait composition. In complex habitats best represented were snails with long reproductive period, while snails which reach maturity later and have more offspring, were more abundant in less complex habitats (Fig. 5b). Woodland species, species living in woody debris, leaf litter, and root zone and those feeding on detritus (Fig. 5c) responded significantly and positively to habitat complexity, which explained 11% (9.1% adjusted) of the variation in specialization traits (pseudo-F = 5.7, p = 0.001). Species living in tall herb habitats, preferring tree/shrub, herb, and soil microhabitats, non-calcareous soils, and feeding on living vascular plants (Fig. 5c), were significantly and negatively correlated with habitat complexity. The community tolerance trait composition responded best to habitat complexity, which explained 16% (14.2% adjusted) of its variation (pseudo-F = 8.8, p = 0.001). Species that have the ability to survive longer periods of dryness, as well as species that prefer arid habitats, were correlated with less complex habitats (Fig. 5d).

Litter was the most important habitat complexity component influencing snail trait composition. It was included as significant predictor of all of the four trait groups, and for three of the groups (morphology, reproduction, and tolerance for habitat conditions) it was the only significant predictor (Table 3). Larger snails with oblong shells were associated with less litter (Appendix A, Fig. S2a), while snails with longer reproduction period, self-fertilizing, with few offspring and early maturation prevailed in habitats with higher abundance of litter (Appendix A, Fig. S2b). Abundance of logs had a stronger effect on specialization traits than litter (Table 3). Open habitat snails, those preferring grassland, herbs, and tall herbs, feeding on live and dead vascular plants, were significantly less abundant in habitats with more logs, where species preferring woods, woody debris, and leaf litter and feeding on litter, lichens, and algae were predominant (Appendix A, Fig. S2c), showing a significant positive response. Detritus feeders were more abundant in habitats with high litter abundance, while snails preferring trees/shrubs and non-calcareous substratum showed an opposite trend (Fig. S2c), their responses being significant. Increased abundance of litter was also associated with higher tolerance to inundation, humidity preference, and lower ability to survive dry periods (Appendix A, Fig. S2d), but only the last two traits showed a significant response.

**Table 3:** Parameters of the best models explaining the snail trait compositions for the four groups of traits and the simple and conditional effects of the included predictors. The p-values for the predictors are adjusted using the false discovery rates.

Predictors	Simple effects				Conditional effects		
	pseudo-F	p	Explained variation (%)	Adjusted explained variation (%)	pseudo-F	p	Explained variation (%)
Morphology							
Litter	9.4	0.002	16.9	15.1	9.4	0.002	16.9
Reproduction							
Litter	9.1	0.002	16.5	14.7	9.1	0.002	16.5
Habitat, microhabitat, soil, and food specialization							
Logs	7	0.001	13.2	11.3	7	0.001	13.2
Litter	5.1	0.001	10.1	8.1	2.7	0.032	4.9
Model (logs + litter)	5	0.001	18.1	14.4			
Tolerance for habitat conditions							
Litter	9.0	0.002	16.4	14.6	9.0	0.001	16.4

### Functional diversity and habitat complexity

**Figure 6:**
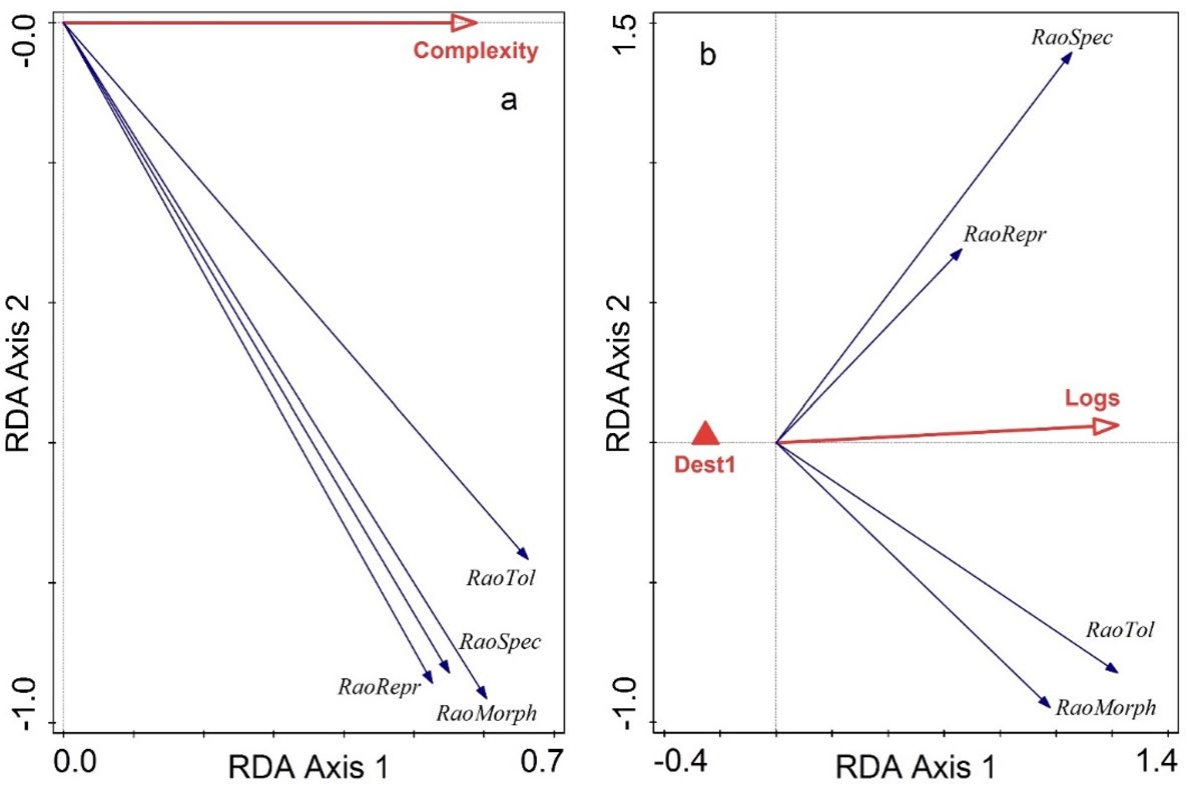
RDA biplot illustrating functional diversities expressed as Rao’s quadratic entropy in relation to: (**a**) habitat complexity and (**b**) its components with a significant effect. Dest1—adjacent fields with agricultural land use. The abbreviations of the four trait categories are given in Table 2.

Habitat complexity also affected significantly the functional diversity of the land snail communities, calculated for the four trait categories: morphology, reproduction, tolerance, and specialization (pseudo-F = 6.2, p = 0.005). Functional diversity increased with habitat complexity (Fig. 6a), which explained 11.9% (10% adjusted) of the variation in the functional diversities, all the responses being significant.

Among the complexity components, the amount of logs was positively correlated with the functional diversity, while the agricultural destination of the surrounding fields was associated with its low values (Fig. 6b). These predictors explained 24.1% (20.7) of the variation in functional diversity (pseudo-F = 4.1, p = 0.003).

## Discussion

We studied the way differences in habitat complexity are reflected in land snail species diversity, trait composition, and functional diversity. This is the first study analyzing snail traits and functional diversity in riparian forests in relation to habitat complexity.

The effect of habitat complexity on land snail communities was the subject of several studies in grasslands^26–28^ and forests^29^. Their results are consistent with the habitat heterogeneity hypothesis^4^, suggesting that structurally complex habitats provide ecological niches and resources for a wide variety of species, thus the larger diversity exhibited in more complex habitats.

Our results confirm the findings of previous studies, riparian forest complexity proving a powerful predictor of the land snail species richness and abundance. In riparian forests, the more complex vertical stratification increases the shade, preserves humidity, and generates litter and dead wood, favoring the presence of snails. The importance of leaf litter and woody debris microhabitats for forest snails was documented by many studies [e.g.,^36,44–47^]. Many snails, especially micro snails, are litter dwellers, while other species use coarse woody debris to shelter from climatic extremes and feed on bacteria, algae, and fungi growing on decaying wood.

Additional ecological niches are generated by the presence of terraces, components of the riparian landscape, generally associated with deeper riverbeds and steep slopes, where land snails can find shelters, being less exposed to insulation and flooding. Among the snail species best responding to habitat complexity were *V. gulo, I. isognomostomos, B. fallax*—preferring decaying wood microhabitats—and minute species living in leaf litter.

These results are confirmed by the trait composition, also significantly affected by the habitat complexity. In more complex habitats, woodland snails, snails living in woody debris, leaf litter, and root zone and those feeding on detritus are more abundant.

On the contrary, species preferring trees/shrubs, herbs, soil microhabitats, noncalcareous soil, and feeding on living vascular plants, are more abundant in less complex habitats. Two species were responding negatively to habitat complexity, *H. lutescens* and *C. vindobonensis*. Both are open habitat snails, living mostly in tall herbs growing at the edge of the riparian forest. These tall herbs, lining the riparian forest in many areas, are generally represented by exotic invasive plant species such as *Helianthus decapetalus* and *Solidago canadensis*, but also by some native species such as *Cirsium oleraceum, Telekia speciosa, Petasites hybridus*. In disturbed forests, as an effect of deforestation, the vertical structure is altered, the amount of litter decreased, and the decaying wood absent, as the old willows were already cut. The tall herbs from the forest edge spread into the riparian forest and occupy the space remaining after logging, explaining the presence of *H. lutescens* and *C. vindobonensis* inside the remnant forest, along with the decrease in habitat complexity. The impact of alien plant species on snail communities was the subject of several studies reporting rather contrasting responses. Some authors have reported a decrease in snail abundance and species richness in invaded plots^48^, while others have found increased species richness and abundance^49^. Our previous study had not shown any effect of allochthonous invasive plant species on riparian snail communities^36^. The response of land snail communities to exotic species seem to depend on the invasive plant species, more probably indirectly affecting the snails, through changes in vegetation structure and subsequent changes in microclimate^50^.

Morphology, tolerance, and reproduction traits are also responding to habitat complexity. The negative response of large snails, reaching later sexual maturity and having more offspring, is most probably explained by the preference of large helicid snails for less complex habitats. The number of eggs (and by default the number of offspring) is known to be small in minute species with short lifespans occurring in leaf litter^51–53^. Baur^51^ issues two hypotheses concerning this convergence: it can be the result of specific natural selection processes in the leaf litter microhabitat or, a general constraint caused by the very small size of the animals. Clausiliid species are within the typical range of medium-sized snails and their batches usually range from one to about a dozen eggs, less in the ovoviviparous and egg-retaining species, as is the case of *Vestia gulo*^54^. Meanwhile, helicid snails, especially the large ones, produce many eggs (over 90 for large species as *Helix pomatia* [Dziabaszewski, 1975 ap^55^]).

A drop in complexity, especially regarding the vertical structure of the vegetation, leads to a change in the water regime, favoring the snails preferring arid habitats and having the ability to survive longer periods of dryness.

The positive response to habitat complexity of snails with longer reproductive periods could be explained by the more stable conditions in microhabitats such as leaf litter, root zone, and decaying wood, allowing reproduction during a longer period.

Functional diversity of riparian snail communities was positively correlated with habitat complexity for all four trait categories. Complex habitats shelter more diverse snail communities, both in terms of species and trait composition. The relationship between taxonomic and functional diversity has been found to take various forms, from the increasing linear relationship most often reported, to a saturation relationship, or even a negative one^56,57^. Habitat complexity was reported to promote functional diversity in snails also at intraspecific level, by allowing the coexistence of individuals with different shell morphology^58^.

Among the complexity components, the amount of logs and the adjacent agricultural fields were correlated with the functional diversity. The presence of dead wood significantly increased the functional diversity of the snail community. Surrounding habitats can contribute with species to the riparian snail community, the contribution being more significant when the two involved habitats are similar, as in the case of riparian forest and other remnant forest patches^36^. Ruderal lands, with bushes and tall herb vegetation that settles there, can also represent favorable habitats for some species of snails, extending the area with suitable conditions beyond the forest edge. The importance of tall herb habitats for overall snail abundance in the study area is confirmed by snail habitat preferences (Fig. 2a). Nevertheless, no association was found between adjacent forest and ruderal lands, and taxonomic or functional diversity. The only relationship was found between the neighboring agricultural land and low functional diversity of land snail communities. In agricultural areas, especially in areas where suitable agricultural land is scarce (as in the hilly areas where our study was conducted), there is an increased pressure on riparian forests because most agricultural land is located along rivers where the soil is more fertile. Expansion of agricultural land through logging results in the narrowing of forests and disappearance of large old trees. Less litter and logs accumulate in the remaining vegetation, decreasing the diversity of the snail community. The presence of old trees is evidence of temporal continuity of the forest and is known to significantly contribute to snail diversity^36,59^.

To conclude, all our hypotheses were confirmed. Our study shows that habitat complexity has a major effect on land snail community structure. The riparian landscape, abiotic conditions, and biotic interactions act as filters, selecting individuals based on their traits. The main components in habitat complexity affecting snail assemblages are the litter and decaying wood, which are the most important microhabitats for riparian forest snails. Additionally, the topographic heterogeneity (the presence of terraces), a good vertical vegetation structure, shading and keeping moisture, and the limitation of human impact (reflected by the agricultural use of the adjacent fields), contributes to shaping the snail communities.

We showed that the loss of habitat complexity, as a result of increased anthropic activities and pressures on riparian forest leads not only to a change in the snail community species composition and reduction of species richness, but also to a shift in the functional composition of the communities and loss of functional diversity, with potential negative effects on the ecosystem services delivered by these natural habitats.

## Supplementary Information

Supplementary Information 1.

Supplementary Information 2.

## Data Availability

All data generated or analysed during this study are included in this published article and its supplementary information files.
