# Supplementary material for: Taxonomic and functional diversity of land snails reflects habitat complexity in riparian forests
Source: Sci Rep. 2023 Jun 17;13:9808. doi: 10.1038/s41598-023-36896-6 (PMC10276844; doi:10.1038/s41598-023-36896-6)
Supplement: Supplementary file 1 — Supplementary Information 1. [file 41598_2023_36896_MOESM1_ESM.docx]

Appendix A.

Table S1. Location of the sampling points and parameters used to evaluate the habitat complexity: total tree cover (trcov), shrub cover (shrb), tall herb cover (tallherb), total herb cover (herb), liana cover (liana), number of tree species (notrsp), abundance of decaying wood (logs) and litter (litter), presence of terraces (terrace), destination of adjacent fields (adfdest). For each sampling site latitude (lat), longitude (long) and altitude (alt, expressed in m) is given. The last column represents the complexity value for each sampling station.

| **No** | **Locality** | **lat** | **long** | **alt** | **trcov** | **shrb** | **tallherb** | **herb** | **liana** | **notrsp** | **logs** | **litter** | **terrace** | **adfdest** | **Compl** |
| --- | --- | --- | --- | --- | --- | --- | --- | --- | --- | --- | --- | --- | --- | --- | --- |
| 1 | **Bârghis 1** | 24.519 | 46.009 | 467 | 2 | 2 | 1 | 2 | 0 | 2 | 1 | 1 | 0 | 0 | **11** |
| 2 | **Bârghis 2** | 24.519 | 46.016 | 466 | 3 | 1 | 0 | 2 | 2 | 4 | 2 | 3 | 1 | 0 | **18** |
| 3 | **Richiș 1** | 24.480 | 46.086 | 468 | 3 | 3 | 0 | 3 | 2 | 3 | 1 | 1 | 2 | 1 | **19** |
| 4 | **Richiș 2** | 24.497 | 46.120 | 409 | 3 | 0 | 3 | 1 | 0 | 2 | 1 | 1 | 0 | 1 | **12** |
| 5 | **Biertan 1** | 24.508 | 46.126 | 393 | 2 | 2 | 3 | 1 | 0 | 2 | 0 | 1 | 0 | 1 | **12** |
| 6 | **Biertan 2** | 24.539 | 46.173 | 367 | 3 | 3 | 0 | 2 | 0 | 2 | 1 | 2 | 1 | 0 | **14** |
| 7 | **Valchid 1** | 24.575 | 46.157 | 355 | 3 | 2 | 0 | 2 | 0 | 3 | 2 | 3 | 2 | 0 | **17** |
| 8 | **Valchid 2** | 24.601 | 46.210 | 361 | 3 | 3 | 0 | 2 | 0 | 3 | 0 | 1 | 1 | 0 | **13** |
| 9 | **Copșa Mare** | 24.563 | 46.149 | 385 | 2 | 0 | 3 | 3 | 0 | 4 | 1 | 3 | 0 | 0 | **16** |
| 10 | **Laslea** | 24.640 | 46.190 | 376 | 2 | 1 | 3 | 2 | 0 | 3 | 1 | 1 | 1 | 0 | **14** |
| 11 | **Roandola 1** | 24.613 | 46.169 | 388 | 2 | 1 | 1 | 3 | 0 | 2 | 1 | 1 | 0 | 1 | **12** |
| 12 | **Roandola 2** | 24.606 | 46.161 | 392 | 3 | 2 | 2 | 3 | 0 | 6 | 2 | 1 | 2 | 2 | **23** |
| 13 | **Mălâncrav 1** | 24.644 | 46.166 | 410 | 2 | 2 | 1 | 2 | 0 | 4 | 1 | 0 | 2 | 0 | **14** |
| 14 | **Mălâncrav 2** | 24.640 | 46.132 | 452 | 2 | 2 | 0 | 2 | 0 | 2 | 1 | 0 | 0 | 0 | **9** |
| 15 | **Iacobeni** | 24.667 | 46.085 | 488 | 2 | 2 | 2 | 3 | 1 | 2 | 2 | 1 | 0 | 0 | **15** |
| 16 | **Hoghilag** | 24.631 | 46.218 | 349 | 2 | 2 | 2 | 2 | 0 | 3 | 0 | 1 | 0 | 1 | **13** |
| 17 | **Daneș** | 24.704 | 46.196 | 383 | 3 | 3 | 3 | 2 | 0 | 3 | 2 | 3 | 2 | 3 | **24** |
| 18 | **Criș** | 24.706 | 46.191 | 395 | 0 | 3 | 0 | 1 | 0 | 2 | 1 | 3 | 1 | 2 | **13** |
| 19 | **Stejăreni** | 24.708 | 46.166 | 422 | 3 | 2 | 3 | 1 | 0 | 2 | 2 | 2 | 0 | 2 | **17** |
| 20 | **Saschiz** | 24.971 | 46.207 | 417 | 1 | 2 | 0 | 3 | 0 | 2 | 1 | 0 | 0 | 0 | **9** |
| 21 | **Meșendorf** | 25.018 | 46.100 | 491 | 2 | 2 | 1 | 1 | 0 | 2 | 1 | 0 | 1 | 1 | **11** |
| 22 | **Bunești** | 25.059 | 46.089 | 533 | 3 | 2 | 0 | 1 | 0 | 2 | 2 | 3 | 0 | 1 | **14** |
| 23 | **Sighișoara** | 24.764 | 46.177 | 393 | 2 | 1 | 1 | 1 | 0 | 1 | 0 | 0 | 0 | 1 | **7** |
| 24 | **Șaeș** | 24.802 | 46.145 | 446 | 2 | 2 | 0 | 2 | 0 | 2 | 2 | 1 | 0 | 0 | **11** |
| 25 | **Apold** | 24.868 | 46.130 | 472 | 2 | 2 | 1 | 1 | 2 | 3 | 1 | 1 | 0 | 0 | **13** |
| 26 | **Retiș** | 24.843 | 46.067 | 483 | 2 | 0 | 1 | 1 | 0 | 2 | 0 | 0 | 0 | 1 | **7** |
| 27 | **Netuș** | 24.779 | 46.050 | 465 | 2 | 1 | 1 | 1 | 0 | 2 | 0 | 0 | 0 | 0 | **7** |
| 28 | **Ucea** | 24.639 | 45.785 | 408 | 2 | 3 | 1 | 1 | 0 | 2 | 0 | 2 | 0 | 2 | **13** |
| 29 | **Cincșor** | 24.833 | 45.848 | 449 | 2 | 2 | 1 | 1 | 1 | 3 | 1 | 1 | 0 | 0 | **12** |
| 30 | **Dacia** | 25.114 | 46.052 | 497 | 2 | 2 | 1 | 2 | 0 | 2 | 1 | 1 | 1 | 1 | **13** |
| 31 | **Jibert** | 25.052 | 46.008 | 497 | 2 | 10 | 2 | 2 | 0 | 3 | 1 | 1 | 0 | 0 | **21** |
| 32 | **Lovnic** | 24.983 | 45.971 | 504 | 3 | 2 | 1 | 1 | 0 | 2 | 1 | 1 | 0 | 2 | **13** |
| 33 | **Bărcut** | 24.921 | 46.008 | 525 | 2 | 2 | 2 | 1 | 0 | 2 | 0 | 1 | 0 | 0 | **10** |
| 34 | **Ighiș** | 24.568 | 45.962 | 460 | 2 | 2 | 0 | 3 | 0 | 2 | 0 | 0 | 0 | 1 | **10** |
| 35 | **Hosman** | 24.357 | 45.805 | 401 | 2 | 2 | 1 | 3 | 0 | 2 | 0 | 1 | 0 | 0 | **11** |
| 36 | **Cașolt** | 24.314 | 45.790 | 400 | 0 | 2 | 2 | 2 | 1 | 2 | 0 | 1 | 0 | 0 | **10** |
| 37 | **Cornățel** | 24.357 | 45.805 | 402 | 2 | 3 | 0 | 2 | 2 | 2 | 1 | 1 | 0 | 0 | **13** |
| 38 | **Fofeldea** | 24.450 | 45.854 | 418 | 0 | 3 | 1 | 2 | 0 | 2 | 1 | 1 | 0 | 0 | **10** |
| 39 | **Nocrich** | 24.457 | 45.864 | 428 | 2 | 2 | 1 | 2 | 1 | 2 | 1 | 0 | 0 | 0 | **11** |
| 40 | **Marpod** | 24.510 | 45.872 | 438 | 2 | 0 | 0 | 2 | 0 | 2 | 1 | 1 | 0 | 0 | **8** |
| 41 | **Chirpăr** | 24.596 | 45.879 | 435 | 1 | 0 | 0 | 2 | 0 | 2 | 1 | 0 | 0 | 0 | **6** |
| 42 | **Poienița** | 24.570 | 45.809 | 407 | 2 | 0 | 0 | 1 | 0 | 3 | 0 | 0 | 0 | 1 | **7** |
| 43 | **Șapartoc** | 24.850 | 46.202 | 462 | 3 | 1 | 0 | 2 | 0 | 4 | 3 | 3 | 2 | 3 | **21** |
| 44 | **Șercaia** | 25.197 | 45.862 | 443 | 0 | 1 | 0 | 2 | 0 | 2 | 0 | 0 | 0 | 0 | **5** |
| 45 | **Crihalma** | 25.216 | 45.919 | 446 | 3 | 1 | 0 | 3 | 0 | 2 | 0 | 1 | 0 | 1 | **11** |
| 46 | **Ticușu Nou** | 25.179 | 45.924 | 452 | 2 | 3 | 0 | 3 | 1 | 2 | 1 | 1 | 0 | 1 | **14** |
| 47 | **Ticușu Vechi** | 25.109 | 45.927 | 467 | 2 | 2 | 0 | 3 | 2 | 3 | 3 | 2 | 0 | 0 | **17** |
| 48 | **Felmer** | 24.958 | 45.915 | 433 | 0 | 2 | 0 | 2 | 0 | 1 | 0 | 0 | 0 | 0 | **5** |


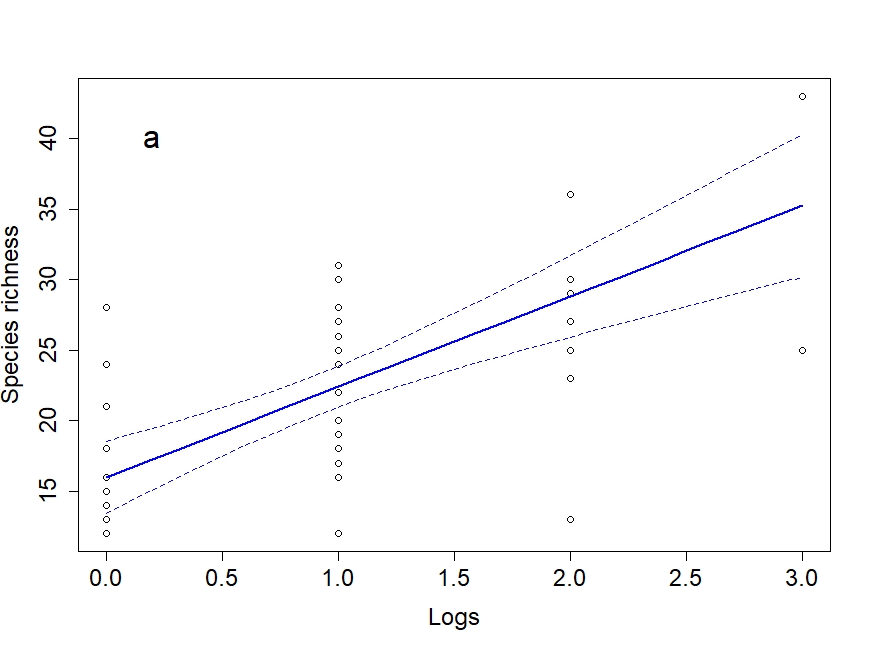

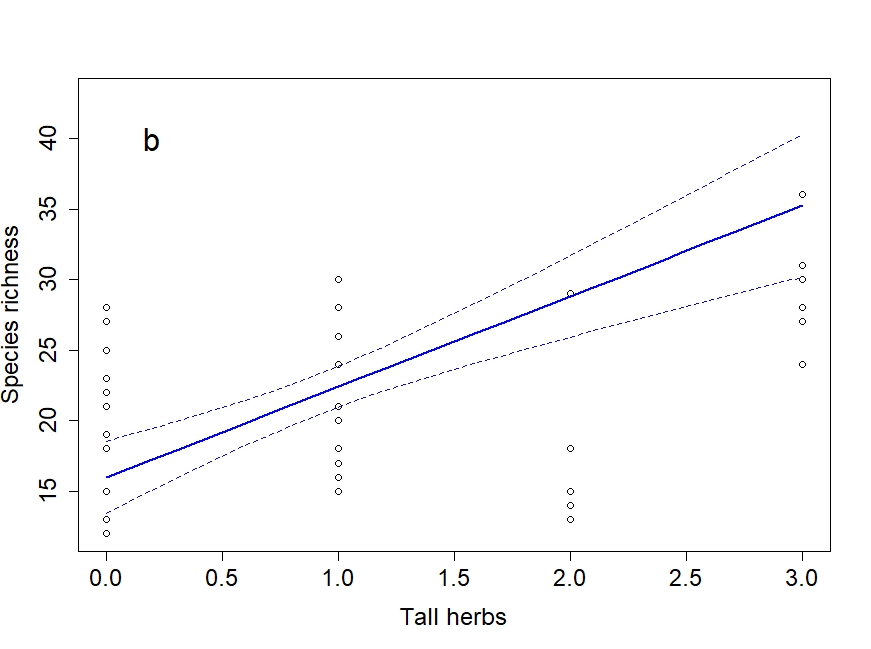


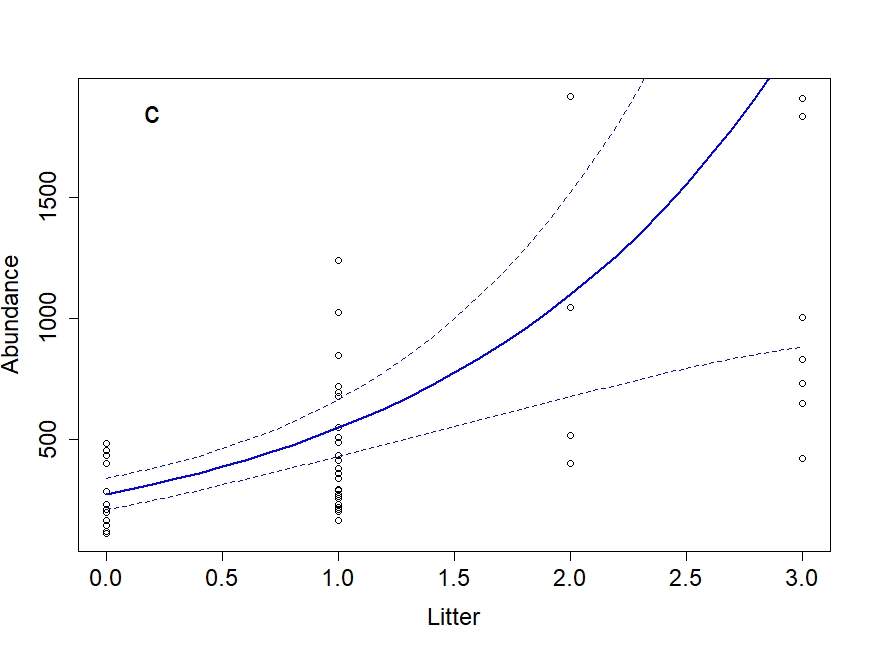

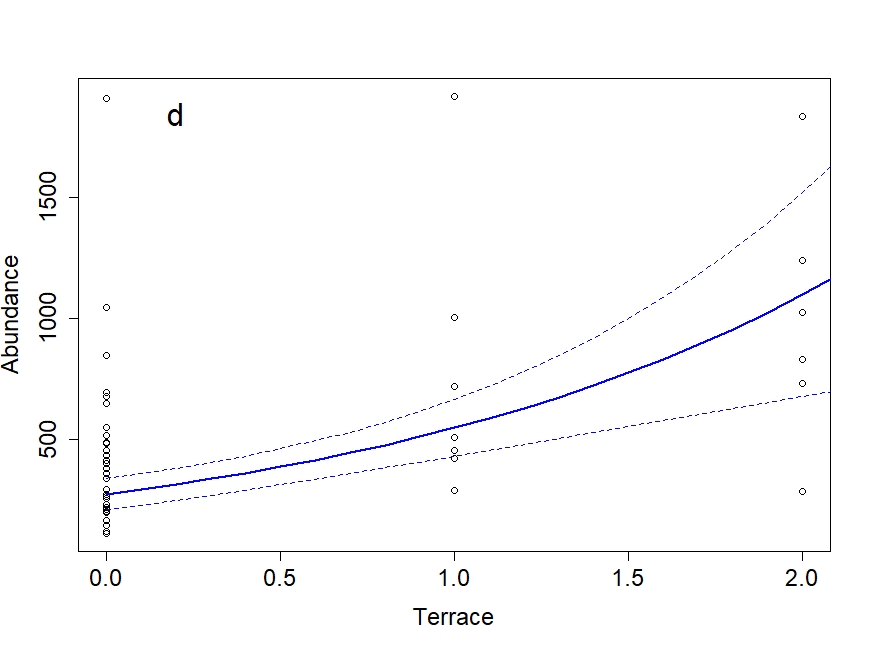


Fig. S1. Responses of community parameters to the habitat complexity components with significant effect – relationships between species richness and a. logs, b. tall herbs and between abundance and c. litter, d. terrace. The 95% confidence interval for the mean estimated value of species richness and abundance is plotted in dotted lines.

| 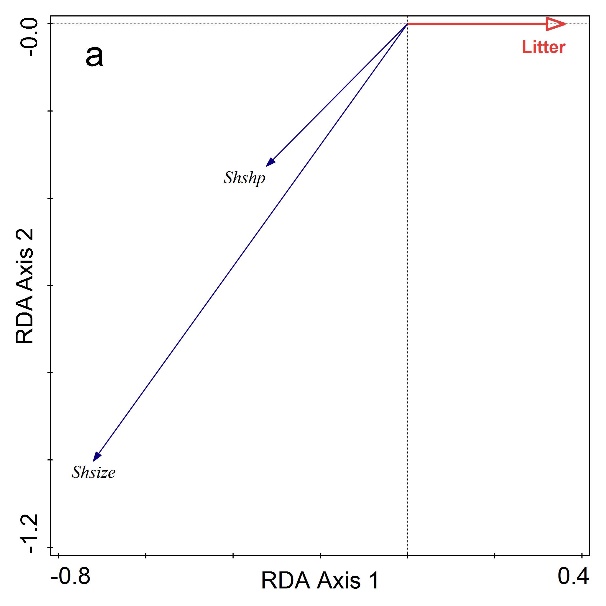 | 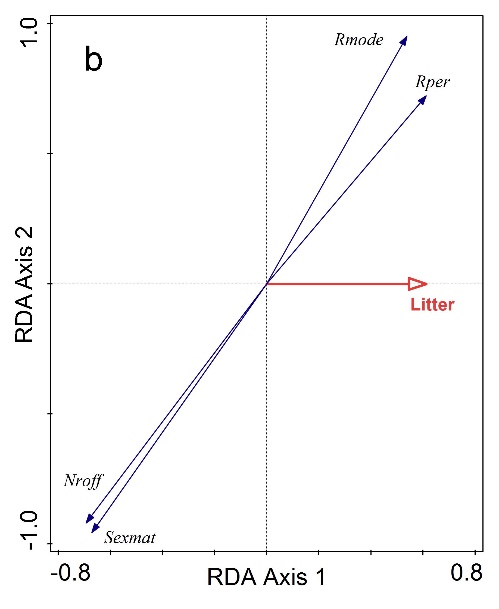 |
| --- | --- |
| 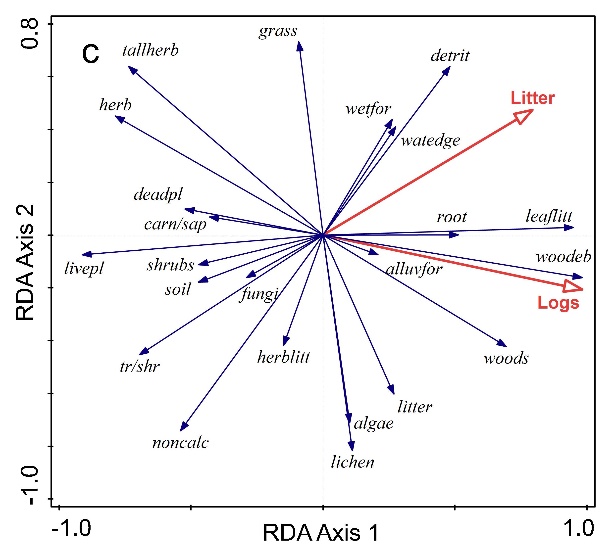 | 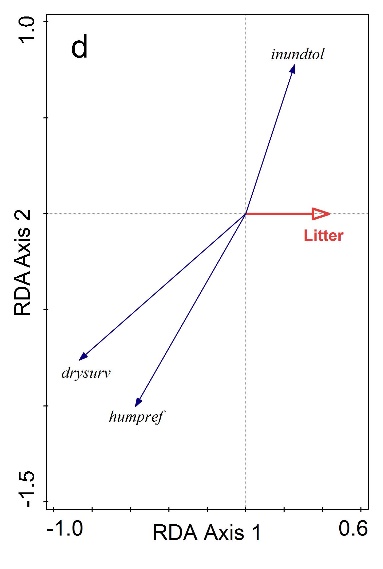 |

Fig. S2. Redundancy analysis (RDA) biplot for the relationships between land snail species community weighted means (CWM) and habitat complexity components with significant effect on traits concerning a. morphology, b. reproduction, c. specialisation and d. tolerance for habitat conditions.
